# Impact of clinical pharmacist-led interventions on denosumab adherence

**DOI:** 10.1007/s11657-026-01725-6

**Published:** 2026-08-06

**Authors:** Anna Zuschnitt, Erin Lightheart, Jennifer Myers, Amna Nabeel Khan

**Affiliations:** 1 Ambulatory Clinical Pharmacy Specialist, Endocrinology, Perelman Center for Advanced Medicine, 3400 Civic Center Blvd, Philadelphia, PA 19104 USA; 2 Department of Quality and Safety, Hospital of the University of Pennsylvania, 3400 Spruce Street, Philadelphia, PA 19104 USA; 3 Clinical Medicine, Perelman School of Medicine, University of Pennsylvania, Center for Healthcare Improvement and Patient Safety, Leonard Davis Institute, Hospital of the University of Pennsylvania, 3400 Spruce Street, Maloney Building, Suite 5003, Philadelphia, PA 19104 USA; 4 Clinical Medicine, Endocrinology, Diabetes and Metabolism, Perelman School of Medicine, University of Pennsylvania, Corporal Michael J. Crescenz VA Medical Center, Philadelphia, USA

**Keywords:** Adherence, Clinical pharmacist, Denosumab (D Mab), Osteoporosis

## Abstract

**Summary:**

Pharmacist-led interventions significantly improved denosumab adherence in patients with osteoporosis at an academic medical center, reducing non-adherence from 20.8 to 4.9%. This quality improvement initiative included pharmacist-led medication counseling and laboratory monitoring, highlighting the effectiveness of pharmacist-physician collaboration in preventing rebound-associated vertebral fractures and enhancing patient outcomes.

**Background:**

Interrupting or delaying denosumab beyond 7 months (210 days) from the last injection leads to transient increase in bone turnover known as rebound phenomenon, resulting in rapid loss of bone density and increased risk of vertebral fracture(s), now known as *rebound-associated vertebral fractures (RAVF)*. Numerous studies suggest that clinical pharmacist participation in chronic disease state management leads to an increase in medication adherence and positive clinical outcomes. We evaluated the impact of a clinical pharmacist intervention on adherence to optimal denosumab timing for patients with osteoporosis.

**Methods:**

The model for improvement was used to design and evaluate this quality improvement project. This study analyzed 143 patients receiving denosumab therapy in an osteoporosis clinic every 6 months for the indication of osteoporosis. At baseline, 21% of patients receiving denosumab did not receive the medication at optimal time intervals for clinical effectiveness. An administration was considered *correct* or on time if denosumab was given within 210 days of the last injection received. If the injection was given outside this window, it was considered *incorrect* administration. The incidence of *correct* and *incorrect* administrations was gathered to calculate the non-adherence rate. Clinical pharmacist interventions included patient counseling and electronic dosing reminders and phone calls. The aim of this study was to reduce the nonadherence rate from 20.8% to less than 5.0% by July 31, 2023.

**Results:**

With these interventions, the number of correct administrations between incorrect administrations improved from an average of 3.3 to 13.1. These effects became visible about 10 weeks after the second intervention and correspond to a reduction in nonadherence rate from 20.8 to 4.9% by July 31, 2023.

**Conclusions:**

Pharmacist-physician collaboration can reduce the nonadherence rate for drugs such as denosumab, interruption of which can result in serious consequences.

## Introduction

Osteoporosisis a chronic asymptomatic metabolic bone disease associated with low bone density, poor bone quality, and reduced bone strength which manifests clinically as a fracture. Fractures related to osteoporosis are frequent. Although one study reported a consistent decline in fracture rate from 2007 to 2013, trends from 2014 to 2017 indicate that fracture rates are no longer declining and for some fracture types, rates are rising [1, 2]. Khan et al. in a recent study report a rising trend in hip fractures in male veterans from 2006to 2019, a finding that underscores the growing concern regarding fracture risk in aging populations and highlights the need for greater attention to osteoporosis at both clinical and public health levels [3].

Denosumab is one of the several medications available that reduce the likelihood of osteoporotic fractures. Denosumab is a humanized monoclonal antibody initially approved by the FDA in 2010 for the treatment of both post-menopausal and male osteoporosis [4]. It is given as a 60-mg subcutaneous injection every 6 months in a health care setting. It is very effective in reducing fractures at all sites; however, numerous published studies indicate that abrupt discontinuation of denosumab leads to a rapid reversal of the bone mineral density (BMD) gains seen with the medication [5, 6]. Further, delaying denosumab doses beyond 7 months results in transient increase in bone turnover known as the rebound phenomenon [7]. Both scenarios increase the risk of multiple vertebral fractures known as *rebound-associated vertebral fractures* (RAVF).

Osteoporotic fractures already impose substantial clinical, functional, and economic burden [8]. Within this context, rebound-associated vertebral fractures can double or triple the per-patient cost of post-fracture management and substantially elevate risks of functional decline and recurrent fractures.

Given the magnitude and preventable nature of this risk, urgent interventions are necessary to reduce avoidable harm and economic waste.

Clinical pharmacists have improved the treatment effect of several chronic disease states such as hypertension and diabetes mellitus, and a few studies suggest that pharmacist involvement in osteoporosis increases the utilization of osteoporosis medications [9–13]. The global aim of this quality-improvement study was to assess the ability of clinical pharmacists to improve adherence rates to denosumab in patients with osteoporosis and possibly prevent transient increase in bone turnover due to unnecessary delays in denosumab therapy.

## Methods

### Setting

This study was conducted at one of the four clinics of Penn Bone Center. Penn Bone Center comprises a team of expert endocrinologists trained in the diagnosis and treatment of bone disorders including osteoporosis.

In July 2021, 20 hours of clinical pharmacist support was added to the bone clinic staff. The aim of clinical pharmacist presence was to assist in improving health outcomes for patients with osteoporosis by providing comprehensive osteoporosis medication counseling, monitoring patients on osteoporosis therapy, and assisting with clinical initiatives within the bone clinic to improve adherence to osteoporosis therapy. These tasks are completed face-to-face, via telehealth, and telephonically with patients.

For this pilot project, patients from a single physician with the largest number of patients receiving denosumab were included. Patients were excluded from the study analysis if they received the drug outside of the health system and there was no clear documentation of denosumab administration dates within the electronic health record (EHR).

### Planning the interventions

We used Plan-Do-Study-Act (PDSA) cycles to identify the problem, conduct a root cause analysis, develop aims and measures, and design and implement interventions.

Prior to implementing either of the interventions, data was collected for patients who had received denosumab for osteoporosis from January 1, 2021, through December 31, 2021, to discern a non-adherence rate. It was calculated that 20.8% of patients receiving denosumab at a single osteoporosis clinic missed one or more injections putting them at high risk for increased bone turnover and vertebral fractures. A process map of current denosumab workflow was then developed and a root cause analysis was performed on a subset of patients receiving denosumab to determine the underlying causes for the delays in denosumab injections (see Figs. 3 and 4 of Appendix). A chart review was completed for those currently on denosumab therapy who had documented delayed or missed doses of denosumab to assess reason for delays. It was determined that missed doses were most often due to patients forgetting the 6-month dosing frequency of denosumab or lack of reply to outreach from the office about upcoming denosumab doses (see Pareto Chart as Fig. 5 of the Appendix). This finding led to one of the interventions of the quality improvement project, which centered around patient reminders. We investigated the potential for the EHR to generate and deliver automated timed reminders through the patient portal both for blood work and denosumab injection administrations. Blood work included a comprehensive metabolic profile to assess calcium levels and a vitamin D test. It was also essential for these reminders to alert the physician and medical team if patient had not read or responded to these reminders. Unfortunately, at the time, the improvement project was conducted, there was not an automated technological solution that met specifications for the reminders, so a clinical pharmacist intervention was planned instead.

The primary goal of this quality improvement initiative was to decrease the non-adherence rate to denosumab therapy to less than 5% by July 31, 2023. Non-adherence is defined as a delay between two injections of > 210 days (more than approximately 7 months). The first step was to analyze the study population’s injections to see if they were correctly administered. An administration was considered correct or on time if denosumab was given within 210 days of the last injection received. If the injection was given outside this time window, it was considered an incorrect administration.

The incidence of correct and incorrect administrations was gathered and used to create the g-chart (Fig. 2), a type of statistical process control chart used to monitor the number of opportunities between rare events.

The non-adherence rate was then calculated by dividing the total incorrect instances in a period by the total incorrect and correct administrations in that same period. We identified 5% as a reasonable target for improvement for a drug like denosumab with serious consequences of delay in even one dose cycle.

### Interventions

Throughout this project, there were two strategies used to improve adherence to denosumab. The first Plan-Do-Study-Act (PDSA) cycle began in October 2021. This first intervention consisted of a pharmacist-led comprehensive counseling session in conjunction with the physician visit during which denosumab initiation was discussed. The physician introduced the clinical pharmacist during the visit and offered each patient the opportunity to meet with the clinical pharmacist to discuss denosumab further. Patients could decline speaking with a clinical pharmacist prior to initiating therapy if they wished. If a patient agreed to meet with a clinical pharmacist, the session was 20 to 30 minutes in length and occurred in person, over the phone, or via telehealth after the patient’s physician visit. During the comprehensive counseling session, the pharmacist completed a comprehensive medication reconciliation with the patient and reviewed the benefits and risks of denosumab therapy with an emphasis on the importance of adhering to every 6-month dosing frequency. This intervention was only offered to patients who were *new to denosumab therapy*; patients previously prescribed denosumab were not approached for simplicity of the intervention.

The second PDSA cycle began in March 2022. A report was generated within the EHR identifying all patients receiving denosumab for the indication of osteoporosis under the care of the participating physician. Patients receiving denosumab for the indication of malignancy were not included because they follow distinct oncology treatment protocols in oncology clinics. The clinical pharmacists used this report to create an active list of patients on denosumab. The list was used to follow up with patients every 5 months to remind patients to complete laboratory tests (calcium and vitamin D) prior to their next denosumab injection. The pharmacists contacted patients either through the EHR or by telephone. Once labs were completed and reviewed, the pharmacists sent a message to the clinic’s nursing staff and prescribing physician for injection scheduling.

### Study of interventions

Data was collected for all patients prescribed denosumab in this one clinic between July 2021 and July 2023. Dates for each denosumab administration were extracted manually from the EHR and recorded. Additionally, it was noted whether the patient had a comprehensive counseling session with the clinical pharmacist about denosumab prior to initiation of the drug. An injection administration was considered correct if denosumab was administered to the patient within 210 days of the previous dose.

### Analysis

Descriptive statistics were used to describe the patient population receiving denosumab. A statistical process control (SPC) chart was used to analyze the number of days between missed denosumab injections over time. The g-chart is a type of SPC chart that enables a view of the process over time with a greater detection power for rare events compared with conventional binomial-based approaches (*The Health Care Data Guide. 2nd edition*).

At the start of the project, a baseline (preintervention) g-chart was constructed by querying the EHRs for patients who had received at least one denosumab injection between January 1, 2021, and December 31 st 2021.

### Ethical considerations

This study was reviewed by the Hospital of the University of Pennsylvania’s Institutional Review Board and determined to be quality improvement.

## Results

From the data collected, we identified 143 patients as having received at least two injections of denosumab during July 2021 and July 2023 in this one osteoporosis clinic. Three were excluded because they received injections at facilities outside of the health system and the exact dates of denosumab administration were not available within the medical records. Baseline demographics are shown in Table 1.

**Table 1:** Baseline demographics

Demographics	Value
Female, *n* (%)	136 (95%)
Male, *n* (%)	7 (5%)
Age at initiation (years)	71.0 (51.5–94.5)*
Patients counseled by pharmacist (#)	
YES	54
NO**	89

*Mean for the age range with the youngest being 51.5 years and oldest being 94.5 years

**Includes patients already established on therapy as well as new starts who declined counseling

**Fig. 1:**
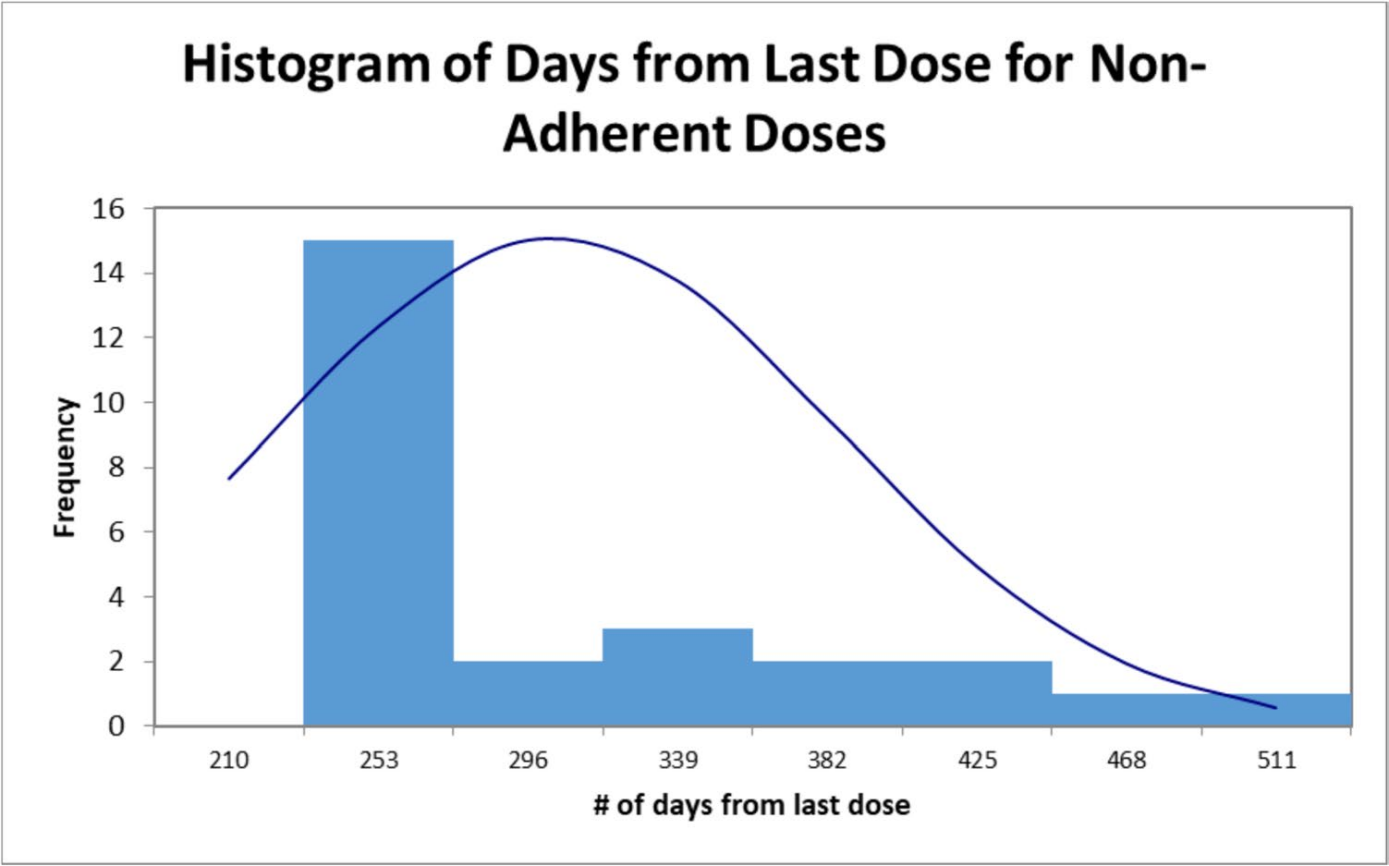
Histogram of days from last dose for non-adherent denosumab doses

The study population’s injections were analyzed to see if they were correctly administered. An administration was considered *correct* or on time if denosumab was given within 210 days of the last injection received. If injection was given outside this time window, it was considered an *incorrect* administration. If denosumab was administered incorrectly or late, the time between the last dose and the delayed dose was recorded during data collection. The average time between delayed doses was 282.7 days with the range of delay from last denosumab dose being 211 to 471 days. A histogram chart (Fig. 1) was created to show the distribution of days elapsed since the previous dose of denosumab. The right-skewed distribution shows that the highest frequency of delayed doses occurred around the 253-day mark.

**Fig. 2:**
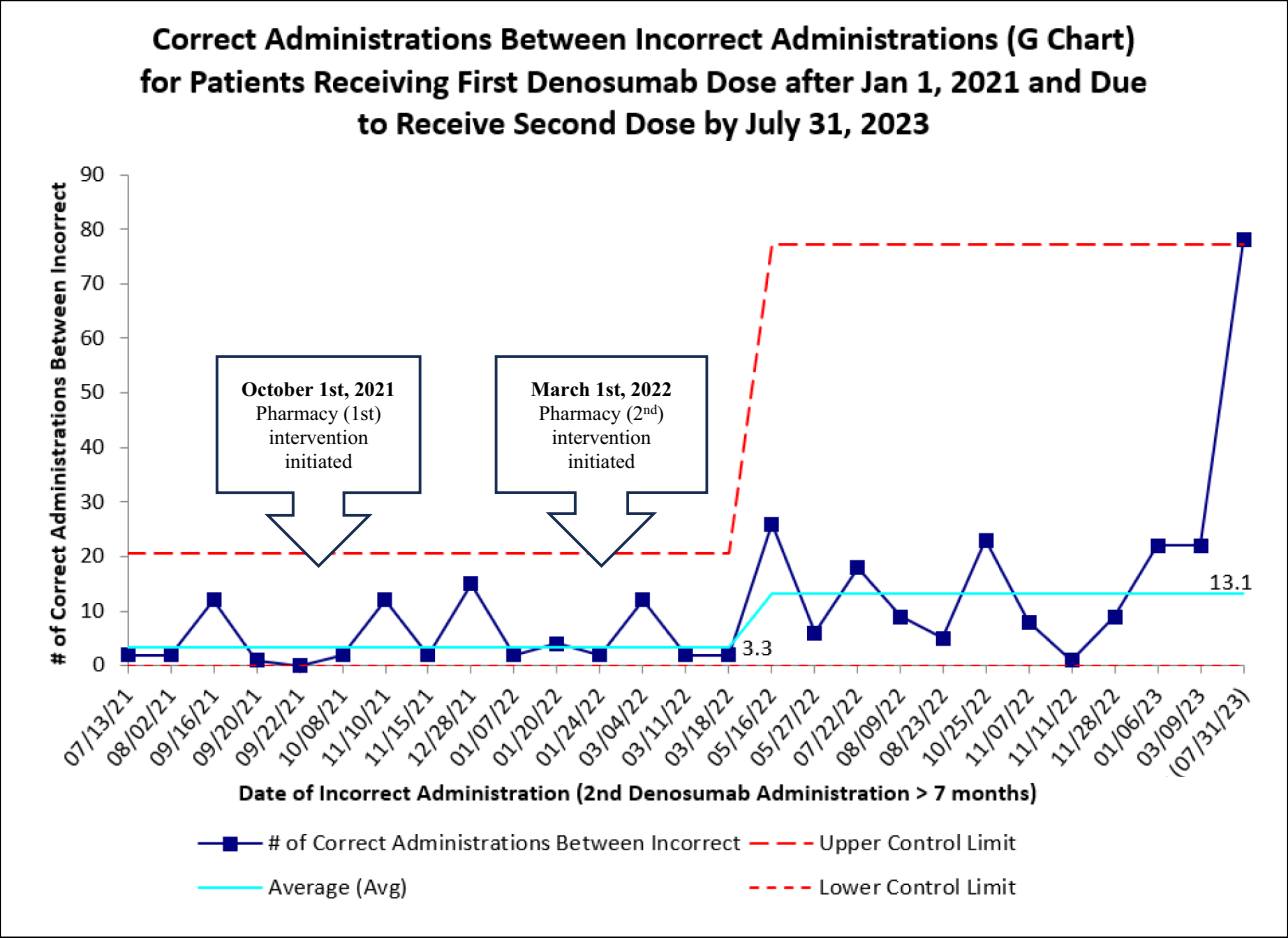
Incidence of correct and incorrect denosumab administrations analyzed as a g-chart

**Fig. 3:**
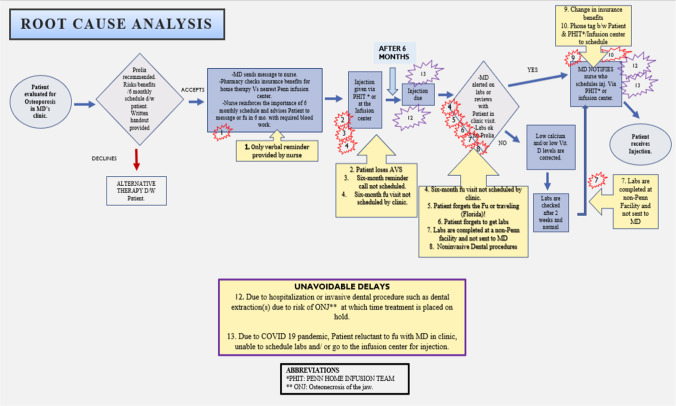
Process map of denosumab administration in bone clinic

**Fig. 4:**
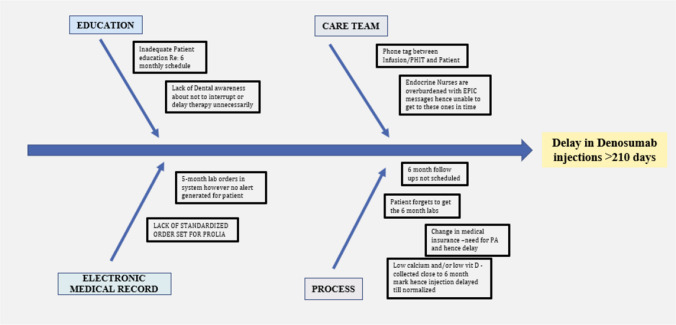
Fish bone analysis

**Fig. 5:**
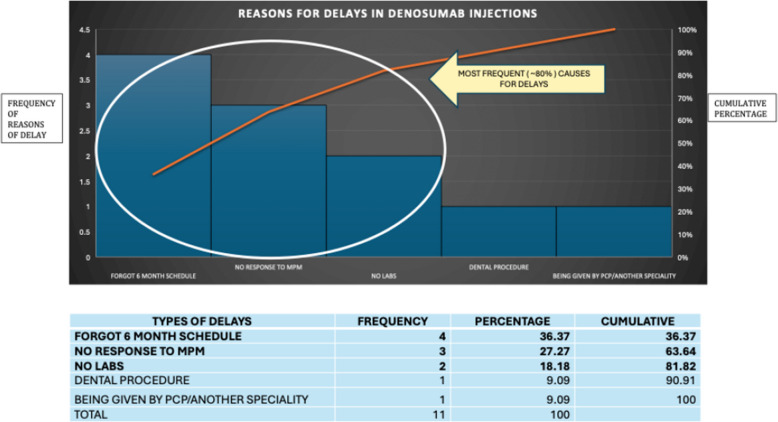
Pareto chart of reasons for delays in denosumab administrations

The incidence of *correct* and *incorrect* administrations was gathered and used to create the g-chart below (Fig. 2).

From the baseline data collected January 1, 2021, to December 31, 2021, the average number of correctly timed denosumab administrations between each incorrect administration was 3.3 visually portrayed in Fig. 2. This means that there were only about three correctly timed or adherent administrations within the clinic in between incorrectly timed or delayed administration. After both interventions were implemented in clinic, the number of correctly timed denosumab administrations in between each inappropriate one increased to 13.1 corresponding with about thirteen correctly timed denosumab administrations within the clinic between each incorrectly timed denosumab administration. Figure 2 also highlights that adherence to denosumab continued to improve as the quality improvement project continued. This is exemplified by the final point on the chart being beyond the upper control limit. This indicates that, at the conclusion of the study period, there were more than eighty correctly timed administrations since the last incorrectly timed or delayed administration of denosumab.

Using the data from the g-Chart in Fig. 2, we calculated the non-adherence rate pre and post both interventions. Prior to pharmacist collaboration, the non-adherence rate was 20.8%. After both interventions were implemented in clinic, non-adherence rate declined to 4.9%, hence within the goal range of < 5%. All participants in the study benefited from the second intervention. Given the 6-month dosing interval of denosumab, this improvement became fully apparent approximately 12 months later (equivalent to two dosing cycles) during PDSA 2.

We also analyzed patients who received counseling by the clinical pharmacist compared to those who either declined the counseling or were already established on the therapy (Table 2 of the Appendix). Non-adherence rate was higher (27%) within the group who were not counseled as compared to 3.7% in the group that received counseling. The difference between these two groups may further emphasize the benefit of having a clinical pharmacist embedded within an osteoporosis clinic, but additional information regarding the reason for a delayed injection may need to be analyzed to prove this finding.

## Discussion

*Rebound-associated vertebral fractures *following denosumab discontinuation are not rare. Numerous published studies have confirmed this serious consequence of abrupt discontinuation or delay in denosumab. A post hoc analysis of the FREEDOM trial and its extension reported significantly higher rates of multiple vertebral fractures in denosumab discontinuation arm as compared to the placebo discontinuation arm [14]. While case reports of non-vertebral fractures after denosumab discontinuation exist, increased risk of such fractures was not seen in the largest retrospective study hence the concern remains mostly for vertebral fractures [15]. *Rebound-associated vertebral fractures* can double or triple the per-patient cost of post-fracture management and substantially elevate risks of functional decline and recurrent fractures. Given the magnitude and preventable nature of this risk, urgent interventions are necessary to reduce avoidable harm and economic waste.

Further, bone turnover data in studies prior to the FREEDOM extension trial post hoc analysis publication indicated that the bone turnover markers, C-terminal cross-linking telopeptide (CTX) and procollagen type 1 N-terminal propeptide (P1NP) increase above baseline values within 3 to 6 months of denosumab discontinuation. Although maximal rebound elevations are often documented closer to 9 to 12 months after the last injection and may present clinically as vertebral fracture(s), the available evidence supports that the rebound process is already biologically underway by approximately 6 to 7 months after the last dose [16, 17]. This quality improvement project was designed to detect delayed denosumab doses that were administered seven months (210 days) or longer after the previous injection in order to prevent the biologic transition toward rebound bone turnover that may begin as early as six to seven months after the last denosumab dose.

Several published studies suggest alternate osteoporosis medications such as bisphosphonates to prevent the rebound bone loss and vertebral fractures; however, very few studies provide strategies for denosumab persistence and address the social barriers to safe use of this therapy.

In this quality improvement study, the feasibility and impact of clinical pharmacist interventions on denosumab persistence are described. With the addition of clinical pharmacist support to the bone clinic, the number of correct administrations of denosumab between incorrect ones improved from an average of 3.3 to 13.1. These effects became visible approximately 10 weeks after the pharmacists began to remind patients of their injection dates and need for pre-injection lab testing. This translated into a reduction in non-adherence rate from 20.8 to 4.9% which was fully apparent 12 months later during PDSA 2.

Another study compares a model where the prescriber maintains responsibility for adherence (denosumab is purchased and dispensed by patient’s own community pharmacy and administered through a home care service) or an all-in-one model where the pharmacist maintains responsibility for the adherence (denosumab is purchased, dispensed, and administered by a pharmacist) [18]. The number of denosumab injections, follow-up appointments on time, denosumab administrations delayed to a maximum of 60 days and the number of denosumab discontinuations were counted. Transfer of complete supply chain to one pharmacy-initiated home care provider resulted in a significant improvement in adherence for denosumab injections as well as significantly lower rates of delayed injections (more than 60 days).

A recent study by Yang Q. et al. reported a significant improvement in proportion of days covered (PDC) value of denosumab with clinical pharmacist adherence management system (CPAMS) [19]. The intervention included automatic reminders to the patient and clinical pharmacist’s work-related mobile phones one week before the injection was due as well as two days later in case the patient did not visit the clinic for the injection, which would prompt the pharmacist to contact patient or their family via telephone.

The role of pharmacists in management of chronic diseases in various specialties is rapidly evolving [18]. Numerous studies have highlighted the significant impact of clinical pharmacists in improving chronic disease outcomes in conditions such as hypertension, diabetes mellitus, and chronic pain management [9, 10, 20].

There is growing evidence that pharmacist involvement in osteoporosis care pathway enhances the utilization of evidence-based medication management. In a study by Bowers et al., high-risk patients with osteoporosis were significantly more likely to be prescribed medications for osteoporosis if their disease state was being managed by both a primary care physician and a pharmacist as opposed to a primary care physician alone [12]. In this collaborative model, pharmacists underwent additional training in osteoporosis management, including review and interpretation of DXAs (dual-energy X-ray absorptiometry). They independently reviewed DXA results, identified appropriate pharmacologic interventions, and communicated their recommendations via electronic medical record to the collaborating physician. Following physician approval, the pharmacist contacted patients to discuss the DXA findings, provide education, and implement the approved management plan.

Another quality improvement initiative highlighted that clinical pharmacists can positively impact osteoporosis pharmacotherapy initiation rates in patients with a history of atraumatic fractures [13]. In this study, a clinical pharmacist was responsible for reviewing DXA scans after a fracture and initiation of osteoporosis medication with physician approval.

Further, evaluation of a pharmacist-led osteoporosis clinic within a primary care setting improved compliance with osteoporosis management guidelines [21]. More patients were appropriately referred for DXA screening and subsequent pharmacologic and non-pharmacologic therapy.

Another study from Kaiser Permanente Colorado reviewed the effectiveness of a clinical pharmacy-based osteoporosis management service in comparison to patients managed by a decentralized nurse-run osteoporosis program within the health system [22]. This study found that postmenopausal females with a recent fracture were three times more likely to initiate medication for osteoporosis if managed by a clinical pharmacist. The authors observed that the differences in outcomes could partly be explained by the integrated nature of clinical pharmacists in clinics. The close-working relationship between pharmacists, providers, and patients could have influenced the provider and patient acceptance of therapy. The specialized training of clinical pharmacists makes them uniquely qualified to evaluate drug therapy and counsel patients on the risks and benefits of medications, known to improve medication adherence and persistence rates.

Forgetfulness has been identified as one of the leading causes of medication non-adherence especially in an aging population with osteoporosis trying to balance multiple commitments against the responsibility of taking numerous medications. This would be considered unintentional as it is outside the control of the patient [23]. Hence, having a clinical pharmacist in the care team would be very beneficial in reminding patients as well as being the point person to answer medication-related questions that could arise during therapy to continuously enhance patient education.

This project has few limitations. First, although the two pharmacist-led interventions were introduced sequentially, they were implemented in overlapping patient populations, including both new denosumab starts and established users, preventing isolation of the individual effects of each intervention. Second, adherence patterns may have been influenced by external factors, particularly the COVID-19 national emergency, during which many patients were reluctant to attend in-person visits for denosumab administration. This concern is supported by joint guidance issued by ASBMR, AACE, the Endocrine Society, and ECTS on mitigating treatment interruptions, as well as by findings from Chandran et al. showing a decline in denosumab adherence from 75.4% pre-pandemic to 63.9% during the pandemic [24, 25]. However, it was the COVID-19 pandemic that clearly highlighted the need for new care pathways to maintain access to therapies requiring in-person administration, such as denosumab—including the potential use of home-infusion nursing services or alternative delivery infrastructure to reduce treatment interruptions. While the interventions noted in this manuscript showed improvement in adherence rates, it must be noted that this project occurred during the COVID-19 pandemic. The resolution of the pandemic may have also naturally improved adherence rates to denosumab. Third, this quality-improvement project reflects the experience of a single center, which may limit generalizability and similar utilization of a clinical pharmacist in other practice settings. Fourth, only patients with fully documented denosumab administrations in the EHR were included excluding those receiving outside the system which could add a bias towards a more adherent population. Additionally, within the confines of this quality improvement project, targeted non-adherence interventions were not feasible. Instead, workflows were created and implemented to integrate a clinical pharmacist, with the goal of enhancing the clinic’s overall adherence to the recommended denosumab injection frequency. Finally, data collection relied on manual monthly chart review by the clinical pharmacist due to the absence of accurate EHR reporting tools for denosumab administration. This process was time-intensive and intermittently redirected pharmacist workload away from direct patient care activities.

To our knowledge, this is the only study analyzing the impact of a clinical pharmacist integrated in a specialized bone clinic on non-adherence to denosumab. In the time after the study period reported in the results section, there has been no incidence of unnecessary delayed denosumab injections which is further proof of the benefit of these clinical pharmacist interventions. The part-time addition of a pharmacist into the clinic has been well received by nursing staff, as it has alleviated the extra workload associated with conducting multiple patient reminder communications. Anecdotally, patients report high satisfaction to the changes that have been implemented to help improve adherence to denosumab injections. This collaboration has also expanded from a single physician to six physicians within the same practice since the conclusion of the project.

This quality improvement initiative has exemplified the benefits that are reaped when a clinical pharmacist joins an ambulatory clinical team. Often, there are many barriers to justifying the presence of a clinical pharmacist in an ambulatory setting that are not limited to the cost of a pharmacist, limited time for clinical-related activities in a pharmacist’s scope, and the lack of support from the other clinical team members to integrate a pharmacist within their team. Fortunately, these barriers were not present during this quality improvement project. The receptiveness of the clinical team and the aid of the health system pharmacy administration to financially justify clinical pharmacist presence in the practice allowed for clinical pharmacy support within the bone clinic. Additionally, the success of this quality improvement initiative was brought to health system leadership and has helped to justify a full-time pharmacist role within the endocrine practice. In the future, the creation of a formal collaborative practice agreement between the clinic’s physicians and pharmacist will allow the pharmacist to comprehensively manage patients on osteoporosis medications as the clinic continues to grow.

## Conclusions

Pharmacist-physician collaboration can improve the adherence rate for drugs such as denosumab and avoid unnecessary delay or interruption of which can result in serious consequences. This quality improvement project highlights that implementation of clinical pharmacist support within an osteoporosis clinic can help to improve adherence to medications and was used to help justify additional pharmacist support for the practice’s osteoporosis team.

## Appendix

**Table 2:** PDSA 1 Medication counseling only by pharmacist

	**Patients ****counseled**** by pharmacist prior to starting denosumab**	**Patients ****not counseled**** by pharmacist prior to starting denosumab**
Number of patients (*n*)	54	89
Total number of denosumab injections received	108	343
Number of instances of non-adherence (delayed administration of denosumab)	2	24
Non-adherence instance rate per number of injection encounters (%)	1.85%	7.00%
Non-adherence rate per number of patients (%)	3.7%	27.0%
